# How does low-input crop management perform? Economic, environmental and social assessment data on different arable crop management strategies in France

**DOI:** 10.1016/j.dib.2025.112316

**Published:** 2025-11-22

**Authors:** Simon Buresi, Chantal Loyce, Rémy Ballot

**Affiliations:** Université Paris-Saclay, AgroParisTech, INRAE, UMR Agronomie, F-91120 Palaiseau

**Keywords:** Pesticides, Fertilisers, Crop management route, Crop sequence, Expert knowledge elicitation

## Abstract

We analysed public raw data from field surveys and elicited knowledge from agricultural advisors to update data about low-input arable crop management performances in France. This dataset includes information about 26 arable crops in 10 zones covering the entire territory of metropolitan France. We differentiated five crop management strategies, ranging from conventional, which is fully input-reliant farming, to organic farming, through integrated pest management and agroecology. Typical crop management routes are given for each situation and assessed through a set of 30 economic, environmental and social performance indicators. This data could be useful to support public decision making regarding low-input agriculture.

Specifications Table

**Table:** 

Subject	Agronomy and Crop Science
Specific subject area	Multicriteria assessment of arable crop low-input management
Type of data	Table
Data collection	Public data from field surveys were analysed and completed using expert knowledge elicited from agricultural advisors
Data source location	Metropolitan France
Data accessibility	Repository name: Référentiel de performances économiques, environnementales et sociales de pratiques culturales économes en intrants Data identification number: https://doi.org/10.57745/LCA116 Direct URL to data: https://entrepot.recherche.data.gouv.fr/dataset.xhtml?persistentId=doi:10.57745/LCA116
Related research article	None

## 1 Value of the Data

• In the European Union and especially in France, public policies target cuts in the use of agricultural inputs (e.g. pesticides and fertilisers) and thus require generic reference points regarding low-input crop management performances.
• A set of such reference points was collected for France around 2010. It needs to be updated, to account for progress in knowledge about low-input crop management.
• Combining 1) an analysis of public data from field surveys and 2) knowledge elicited from experts, namely agricultural advisors involved in on-farm experiments with low-input crop management systems, we compiled data covering the whole of metropolitan France.
• This data considers five crop management strategies, ranging from conventional agriculture, which is fully reliant on inputs, to organic farming, which prohibits the use of synthetic pesticides and fertilisers.
• We assessed the economic, environmental and social performances of most of arable crops for these five crop management strategies, according to 30 indicators.
• This data could be used by other researchers or public policy makers to support forecast studies about scaling-up low-input agriculture.

## 2 Background

In 2010, Butault et al. [1] published reference points about performances of low-input crop management. This data aimed at supporting the French Ecophyto plan, which targeted to halve the use of pesticides in agriculture. This target remains relevant, but reference points are no longer up to date. Indeed, knowledge have progressed in >10 years, as low-input crop management has been experienced, within the Ecophyto plan framework. Data presented here is an update of the reference points previously released by Butault et al.

## 3 Data Description

The data is composed of five CSV files.

### 3.1 1_crop_management_routes.csv

The file “1_crop_management_routes.csv” contains quantitative descriptions of typical crop management routes [2] associated to each cropping situation considered (i.e. a given crop, cultivated in a given zone, within a given crop sequence type and according to a given crop management strategy). Table 1 presents its content.

**Table 1:** Description of the contents of the file “1_crop_management_routes.csv”.

Variable	Name	Description
1	Crop	Crop considered (as defined in Section 3.1.1.)
2	Zone	Zone considered (as defined in Section 3.1.2)
3	crop_sequence_type	Crop sequence type considered (as defined in Section 3.1.3)
4	crop_management_strategy	Crop management strategy considered (as defined in Section 3.1.4)
5	duration	Number of years of cultivation of the crop considered (1 year for most of crops, except temporary grasslands or forage legumes)
6	composition	Number of species composing the crop considered (1 specie for most of crops, except temporary grasslands or intercrops)
7	plowing	Plowing frequency (0 if never plowed or 1 if systematically plowed)
8	secondary_tillage	Average number of secondary tillage operations
9	other_mechanical_operations	Average number of other mechanical operations
10	double_crop	Frequency of double crop sowing before the crop considered
11	double_crop_type	Type of double crop sown before the crop considered
12	covercrop	Frequency of cover crop sowing before the crop considered
13	covercrop_type	Type of cover crop sown before the crop considered
14	sowing	Sowing frequency of the crop considered (1 time a year for most of crops, except temporary grasslands or forage legumes)
15	sowing_rate	Average sowing rate (kg.ha^-1^ or seed.ha^-1^ depending on the crop)
16	harrow_weeding	Average number of harrow weeding operations
17	hoe_weeding	Average number of hoe weeding operations
18	hand_weeding	Average number of hand weeding operations
19	pesticide_spraying	Average number of pesticides spraying operations
20	localised_spraying	Average number of localised weeding operations
21	fertilisers_spreading	Average number of fertilisers spreading operations
22	dble_crop_fertilisers_spreading	Average number of fertilisers spreading operations on double crop
23	manure_spreading	Average number of manure spreading operations
24	manure_type	Type of manure spread
25	dble_crop_harvest	Average number of double crop harvest operations
26	crop_harvest	Average number of crop harvest operations (1 operation for most of crops, except temporary grasslands or forage legumes)
27	hay	Frequency of hay harvest (forage crops only)
28	silage	Frequency of silage harvest (forage crops only)
29	wrapping	Frequency of wrapping harvest (forage crops only)
30	seed_coating	Frequency of use of coated seed
31	herbicides_TFI	Average herbicides Treatment Frequency Index
32	fungicides_TFI	Average fungicides Treatment Frequency Index
33	insecticides_TFI	Average insecticides Treatment Frequency Index
34	in_furrow_ins_TFI	Average in-furrow insecticides Treatment Frequency Index
35	other_TFI	Average other pesticides Treatment Frequency Index
36	molluscicide_TFI	Average molluscicides Treatment Frequency Index
37	growth_regulator_TFI	Average growth regulator Treatment Frequency Index
38	TFI	Average total Treatment Frequency Index
39	iron_phosphate_bio_TFI	Average iron phosphate biocontrol Treatment Frequency Index
40	tricho_bio_TFI	Average trichogramma biocontrol Treatment Frequency Index
41	copper_sulfate_bio_TFI	Average copper sulfate biocontrol Treatment Frequency Index
42	N_rate	Average nitrogen rate (kg_N_.ha^-1^)
43	dble_crop_N_rate	Average nitrogen rate on double crop (kg_N_.ha^-1^)
44	manure_rate	Average manure rate (t.ha^-1^ or m^3^.ha^-1^)
45	irrigation	Frequency of irrigation
46	irrigation_operations	Average number of irrigation operation
47	irrigation_rate	Average rate of irrigation (mm)
48	dble_crop_yield	Average double crop yield (t.ha^-1^)
49	crop_yield	Average crop yield (t.ha^-1^)

### 3.2 2_crop_parameters.csv

The file “2_crop_parameters.csv” contains information that is specific to each crop considered and needs to be combined with quantitative typical crop management route information to compute indicators values. Table 2 presents its content.

**Table 2:** Description of the contents of the file “2_crop_parameters.csv”.

Variable	Name	Description
1	crop	Crop considered
2	destination	Destination of the crop
3	composition	Number of species composing the crop considered (1 specie for most of crops, except temporary grasslands or intercrops)
4	price_conv_low	Selling price of the crop in conventional farming – lowest price recorded over 2011–2021 (€.t^-1^)
5	price_conv_high	Selling price of the crop in conventional farming – highest price recorded over 2011–2021 (€.t^-1^)
6	price_conv_average	Selling price of the crop in conventional farming – average price recorded over 2011–2021 (€.t^-1^)
7	price_org	Selling price of the crop in organic farming – average price recorded over 2011–2021 (€.t^-1^)
8	exp_N_straw_exp	Nitrogen exportation when straw is exported (kg_N_.t^-1^)
9	exp_N_straw_ret	Nitrogen exportation when straw is returned (kg_N_.t^-1^)
10	lower_heating_value	Amount of heat released by combusting a specified quantity (GJ.t^-1^)
11	seed_cost_cert_conv	Cost of certified seeds in conventional farming (€.unit^-1^)
12	seed_cost_cert_org	Cost of certified seeds in organic farming (€.unit^-1^)
13	seed_cost_farm_conv	Cost of farm-saved seeds in conventional farming (€.unit^-1^)
14	seed_cost_farm_org	Cost of farm-saved seeds in organic farming (€.unit^-1^)
15	seed_cost_unit	Unit employed for seeds cost
16	seed_cert_share	Share of certified seeds considered
17	seed_farm_share	Share of farm-saved seeds considered
18	seeder_type	Type of seeder considered
19	cost_herbicides_TFI	Cost of one unit of herbicides Treatment Frequency Index (€)
20	cost_fungicides_TFI	Cost of one unit of fungicides Treatment Frequency Index (€)
21	cost_insecticides_TFI	Cost of one unit of insecticides Treatment Frequency Index (€)
22	cost_in_furrow_ins_TFI	Cost of one unit of in-furrow insecticides Treatment Frequency Index (€)
23	cost_growth_regulator_TFI	Cost of one unit of growth regulator Treatment Frequency Index (€)
24	cost_molluscicide_TFI	Cost of one unit of moluscicides Treatment Frequency Index (€)
25	cost_iron_phosphate_bio_TFI	Cost of one unit of iron phosphate biocontrol Treatment Frequency Index (€)
26	cost_tricho_bio_TFI	Cost of one unit of trichogramma biocontrol Treatment Frequency Index (€)
27	cost_copper_sulfate_bio_TFI	Cost of one unit of copper sulfate biocontrol Treatment Frequency Index (€)

### 3.3 3_Input_parameters.csv

The file “3_input_parameters.csv” contains information about inputs that is not crop-specific and was involved in computing the values of indicators. Table 3 presents its content.

**Table 3:** Description of the contents of the file “3_input_parameters.csv”.

Variable	Name	Description
1	category	Category of the input considered (fertilisation, fuel or irrigation)
2	input	Input considered
3	cost_low	Purchase cost of the input considered – lowest price recorded over 2011–2021 (€/unit)
4	cost_average	Purchase cost of the input considered – average price recorded over 2011–2021 (€/unit)
5	cost_high	Purchase cost of the input considered – highest price recorded over 2011–2021 (€/unit)
6	unit	Unit of cost considered
7	N_content	Nitrogen content of the input (kg_N_.t^-1^)
8	share_use	Share of fertilisers use
9	indirect_energy_cost	Indirect energy cost of fertiliser production (GJ.kg_N_^-1^)
10	direct_energy_cost	Direct energy cost of irrigation (GJ.mm^-1^)
11	CO2_factor_emission	CO2 emission factors used to calculate GHG (teq CO2) emissions from fertilisation, fuel, irrigation and livestock unit
12	N2O_factor_emission	N2O emission factor used to calculate GHG (teq CO2) emissions from nitrogen fertiliser use

### 3.4 4_Operation_parameters.csv

The file “4_operation_parameters.csv” contains information related to the technical operations involved in computing indicator values. Table 4 presents its content.

**Table 4:** Description of the contents of the file “4_operation_parameters.csv”.

Variable	Name	Description
1	operation	Operation considered
2	description	Details of the operation
3	fixed_cost	Cost of the operation, excluding fuel and labour cost (€.ha^-1^)
4	cost_fuel_incl	Cost of the operation including fuel cost (€.ha^-1^)
5	cost_labour_incl	Cost of the operation including labour cost (€.ha^-1^)
6	cost_fuel_and_labour_incl	Cost of the operation including fuel and labour cost (€.ha^-1^)
7	ha_h	Acreage covered per hour (ha.h^-1^)
8	h_ha	Time required to cover 1 hectar (h.ha^-1^)
9	fuel_cons	Fuel consumption (l.ha^-1^)
10	energy_cost	Energy cost (GJ.ha^-1^)

### 3.5 5_Indicators_values.csv

The file “5_indicators_values.csv” contains the values of indicators describing the economic, environmental and social performances of the cropping situations considered. These values were either pulled from the 1_crop_management_routes.csv file or computed from the four preceding files. Table 5 presents the content of this final file.

**Table 5:** Description of the contents of the file “5_indicators_values.csv”.

Variable	Name	Description
1	crop	Crop considered (as defined in Section 3.1.1.)
2	zone	Zone considered (as defined in Section 3.1.2)
3	crop_sequence_type	Crop sequence type considered (as defined in Section 3.1.3)
4	crop_management_strategy	Crop management strategy considered (as defined in Section 3.1.4)
5	crop_yield	Crop yield (t.ha^-1^), as defined in 1_crop_management_routes.csv
6	herbicides_TFI	Herbicides Treatment Frequency Index, as defined in 1_crop_management_routes.csv
7	fungicides_TFI	Fungicides Treatment Frequency Index, as defined in 1_crop_management_routes.csv
9	insecticides_TFI	Insecticides Treatment Frequency Index, as defined in 1_crop_management_routes.csv
10	other_TFI	Other Treatment Frequency Index, as defined in 1_crop_management_routes.csv
11	TFI	Total Treatment Frequency Index, as defined in 1_crop_management_routes.csv
12	bio_TFI	Biocontrol Treatment Frequency Index, as defined in 1_crop_management_routes.csv
13	total_N_rate	Total nitrogen rate (kg_N_.ha^-1^) as defined in 1_crop_management_routes.csv for crop and double crop: total_N_rate=N_rate+dble_crop_N_rate
14	N_balance	Nitrogen balance (kg_N_.ha^-1^), difference between nitrogen inputs, given in 1_crop_management_routes.csv and outputs, considering nitrogen exportation by yield unit, given in 2_crop_parameters.csv: N_balance=N_rate+manure_rate.N_content−crop_yield.exp⁡_N(strawexporrest)
15	irrigation_rate	Irrigation rate (mm), as defined in 1_crop_management_routes.csv
16	livestock_density	Livestock density (livestock unit.ha^-1^), equal to 0 for non-forage crops
17	milk_production	Milk production (l.ha^-1^), equal to 0 for non-forage crops
18	meat_production	Meat production (kg.ha^-1^), equal to 0 for non-forage crops
19	gross_product	Gross product (€.ha^-1^), production, given in 1_crop_management_routes.csv multiplied by its price, given in 2_crop_parameters.csv: gross_product=crop_yield.price_conv_average(orprice_org) Forage crops gross product corresponds to the sale of hay or silage.
20	gross_product_livestock_incl	Gross product (€.ha^-1^), same as gross_product, but for forage crops, gross product correspond to the sale of milk or meat.
21	variable_cost	Variable cost (€.ha^-1^) corresponding to seeds, fertilisers, manure, pesticides and water. Sum of all input rates, given in 1_crop_management_routes.csv multiplied by their unit costs, given in 3_input_parameters.csv: $variable\_cost=\sum_{input}rate.unit\_cost$
22	mechanisation_cost	Mechanisation cost (€.ha^-1^), sum of all operations frequencies, described in 1_crop_management_routes.csv, multiplied by their cost, given in 4_operation_1_crop_management_routes.csv.csv (fuel and labour were not considered): $mechanisation\_cost=\sum_{operation}frequency.fixed\_cost$
23	livestock_cost	Feed, veterinary and breeding costs (€.ha^-1^).
24	gross_margin	Gross margin (€.ha^-1^), difference between gross product and variable cost: gross_margin=gross_product−variable_cost
25	gross_margin_livestock_incl	Gross margin (€.ha^-1^), same as gross_margin but for forage crops, gross product including milk or meat sale and livestock cost were considered: gross_margin_livestock_incl=gross_product_livestock_incl−variable_cost−livestock_cost
26	net_margin	Net margin (€.ha^-1^), gross margin minus mechanisation cost: net_margin=gross_margin−mechanization_cost
27	net_margin_livestock_incl	Net margin (€.ha^-1^), same as net_margin but for forage crops, gross margin including milk or meat sale and livestock cost was considered: net_margin_livestock_incl=gross_margin_livestock_incl−mechanization_cost
28	total_fuel_cons	Total fuel consumption (l.ha^-1^), sum of all operations frequencies, described in 1_crop_management_routes.csv, multiplied by their consumption, given in 4_operation_1_crop_management_routes.csv.csv: $total\_fuel\_cons=\sum_{operation}frequency.fuel\_cons$
29	total_energy_cost	Total energy cost (GJ.ha^-1^), sum of all operations frequencies, described in 1_crop_management_routes.csv, multiplied by their energy cost, given in 4_operation_1_crop_management_routes.csv.csv, direct energy cost related to irrigation and indirect energy cost related to nitrogen fertilisers production, given in 3_input_parameters.csv: $total\_energy\_cost=\sum_{operation}frequency.energy\_cost+irrigation\_rate.direct\_energy\_cost+N\_rate.indirect\_energy\_cost$
30	total_energy_product	Total energy product (GJ.ha^-1^): production, given in 1_crop_management_routes.csv, multiplied by lower heating value, given in 2_crop_parameters.csv for crop and double crop: total_energy_product=crop_yield.lower_heating_value+dble_crop_yield.lower_heating_value
31	energy_efficency	Ratio of total energy product to total energy cost: energy_efficiency=total_energy_product/total_energy_cost.100
32	number_operations	Number of operations, as described in 1_crop_management_routes.csv
33	working_time	Working time (h.ha^-1^), sum of all operations frequencies, described in 1_crop_management_routes.csv, multiplied by the time required to cover 1 hectare, given in 4_operation_1_crop_management_routes.csv.csv: $working\_time=\sum_{operation}frequency.h\_ha$
34	GHG_emissions	Greenhouse gases emissions (t_CO2 equivalent_/ha) due to fuel consumption, energy use for irrigation and fertilisers production, taking into account emission coefficients given in 3_input_parameters.csv: GHG_emissions=total_fuel_cons.emission_coef+irrigation_rate.emission_coef+total_N_rate.emission_coef
35	GHG_emissions_livestock_incl	Greenhouse gases emissions (t_CO2 equivalent_/ha), same as GHG-emissions, but including livestock GHG emissions for forage crops: GHG_emissions_livestock_incl=GHG_emissions+livestock_density.emission_coef

## 4 Experimental Design, Materials and Methods

### 4.1 Cropping situations described

In our dataset, the performances of low-input crop management are provided at the elementary scale of a cropping situation. By cropping situation, we mean a given crop, cultivated in a given zone, within a given crop sequence type and according to a given crop management strategy. 496 cropping situations are described.

#### 4.1.1 Crops

We considered 26 arable crops. These include major arable crops, which are considered by the “*Pratiques culturales grandes cultures et prairies*” field survey [3] (i.e. soft wheat, durum wheat, barley, triticale, grain corn, silage corn, oilseed rape, sunflower, oilseed flax, fibre flax, protein pea, faba bean, soybean, sugar beet, potatoes and temporary grasslands). We differentiated winter and spring types for barley, protein pea, faba bean and oilseed flax. We also considered additional minor crops, as they could be used as diversification crop or are frequently cultivated in organic farming (i.e. alfalfa, hemp, chickpea, lentil, buckwheat, sorghum and cereal-pulse intercrop).

#### 4.1.2 Zone

We subdivided the territory of metropolitan France into 11 zones, which we considered as homogeneous from an agricultural point of view.

We obtained these 11 zones by classifying metropolitan France’s 96 *départements* (administrative counties) according to 19 variables representing the agricultural characteristics of each one (Table 6).

**Table 6:** Variables used to classify the 96 French metropolitan départements into 11 homogeneous agricultural zones.

Agricultural characteristic	Variable
Production systems	Share of the département’s UAA used for arable crops [4]
	Share of the département’s UAA used for dairy cattle [4]
	Share of the département’s UAA used for beef cattle [4]
	Share of the département’s UAA used for other livestock farming [4]
	Share of the département’s UAA used for monogastric livestock [4]
	Share of the département’s UAA used for mixed crop-livestock farming [4]
Weather conditions	Latitude of the département’s centroid
	Longitude of the département’s centroid
Crop successions	Share of crop sequences including oilseed rape in the département’s arable land area [5]
	Share of crop sequences including corn in the département’s arable land area [5]
	Share of crop sequences including temporary grassland in the département’s arable land area [5]
	Share of crop sequences including cereals and temporary grassland in the département’s arable land area [5]
	Share of crop sequences including root crops in the département’s arable land area [5]
	Share of crop sequences including sunflower in the département’s arable land area [5]
	Share of crop sequences including protein pea in the département’s arable land area [5]
	Share of crop sequences including soybean in the département’s arable land area [5]
	Share of crop sequences including forage legumes in the département’s arable land area [5]
	Share of crop sequences including fiber flax in the département’s arable land area [5]
Pedoclimatic conditions	Best average soft wheat yield [6]

We fed these variables into a Principle Component Analysis (PCA) using the R package FactoMineR 2.1′s PCA() function [7]. The first seven components in this PCA, which account collectively for 88 % of the variance, were fed into the same R package’s HCPC() function (hierarchical clustering on principle components), which yielded our 11 groups.

We set aside the first group, which only included the island of Corsica, and ceased to take it into account as no further data were available.

#### 4.1.3 Crop sequence type

Each of the 11 zones we had outlined could encompass a diverse array of production situations [8]. We assumed that the diversity of production situations in a given zone should be reflected by the diversity of crop sequences observed. For example, in a given zone, crop sequences that include temporary grassland should be used more frequently at medium altitudes or in sub-zones presenting limited potentialities, which specialize in livestock farming, whereas crop sequences that include root crops should be confined to sub-zones which present high potentialities and are specialised in arable crop farming. This is why the dataset differentiated values according to crop sequence typology. This typology is based on a classification of crop sequences drawn from the French LPIS [5], according to the method proposed by Ballot et al. [9]. First, for each crop sequence, we calculated temporal frequencies over the period 2015–2019 of 14 (groups of) crops considered as typical of production situations: soft wheat, durum wheat, barley, triticale, corn, oilseed rape, sunflower, soybean, pea, sugar beet, potatoes, fibre flax, temporary grasslands, forage legumes.

We used these frequencies as input variable in a PCA [9]. The 10 first components, which account together for 85 % of the variance, were used as input of a hierarchical clustering differentiating 10 groups of crop sequence, as presented in Table 7.

**Table 7:** Crop sequence types identified, spatial frequency at the scale of France and average temporal frequency of dominant (groups of) crops within the crop sequence.

Crop sequence group	Spatial frequency at the scale of France over the period 2015–2019	Average temporal frequencies of dominant (groups) of crops within the crop sequence
Crop sequence with oilseed rape	25 %	Soft wheat: 0,4 Rapeseed: 0,2 Barley: 0,2
Crop sequence with corn	14 %	Corn: 0,61 Soft wheat: 0,26
Corp sequence with temporary grassland	12 %	Temporary grassland: 0,7
Crop sequence with cereals and temporary grassland	12 %	Triticale: 0,3 Corn: 0,2 Soft wheat: 0,18 Temporary grassland: 0,16
Crop sequence with root crops	10 %	Soft wheat: 0,46 Sugar beet: 0,21 Potatoes: 0,09
Crop sequence with sunflower	7 %	Durum wheat: 0,37 Sunflower: 0,26 Soft wheat: 0,12
Crop sequence with protein pea	6 %	Soft wheat: 0,41 Protein pea: 0,21 Barley: 0,15 Oilseed rape: 0,11
Crop sequence with soybean	5 %	Soybean: 0,28 Soft wheat: 0,28 Corn: 0,27
Crop sequence with forage legumes	4 %	Forage legumes: 0,63 Soft wheat: 0,13
Crop sequence with fiber flax	3 %	Soft wheat: 0,47 Fibre flax: 0,21 Sugar beet: 0,08 Potatoes: 0,05

#### 4.1.4 Crop management strategy

We followed the typology proposed by Butault and al. [1] and Jacquet et al. [10] to identify five crop management strategies (Table 8). Based on the Efficiency-Substitution-Redesign framework [11], this typology remains accurate to differentiate crop management strategies according to their degree of reliance on pesticides or synthetic inputs in general.

**Table 8:** Crop management strategies identified.

Mean	Average conventional farming practices, according to public statistics
N0	Crop management strategy based on a systematic use of synthetic pesticides and fertilisers
N1	Crop management strategy based on an efficient use of synthetic pesticides and fertilisers, thanks to scouting and decision support tools
N2a	Crop management strategy based on Integrated Pest Management (IPM) principles, which includes substitution by non-chemical methods (e.g. mechanical weeding) or redesigned crop management routes to prevent pest damages
N2c	Crop management strategy based on agroecology principles [12], which includes cropping systems diversification to promote natural means of pest regulation
N3	Crop management strategy based on organic farming specifications, which excludes the use of synthetic pesticides and fertilisers.

### 4.2 Data collection

#### 4.2.1 Analysis of public data from field surveys

We analysed raw data from the “*Pratiques culturales grandes cultures et prairies*” field survey to complete the “Average conventional” and “N3” levels. This survey is carried out every five years by the French Ministry of Agriculture to monitor agricultural public policies. The latest campaign available when we produced this dataset concerned the cropping year 2017. Information from around 25,000 fields were collected during in-person interviews with farmers, according to a sampling plan designed to ensure representativeness at the national scale and at the scale of administrative regions. The information collected includes all technical operations carried out on surveyed fields, from the harvest of the preceding crop until the crop’s harvest. This includes tillage, sowing, fertilisation, crop protection, with details about the date of the operation and the nature and amounts of the inputs it involved. Basic information about the five preceding years, including the crop sequence, was also collected.

We assigned each field surveyed to a zone, as defined in Section 3.1.2, according to its location. We also assigned a crop sequence type, as defined in Section 3.1.3, according to the observed crop sequence. We tried to assign a crop management strategy, as defined in Section 3.1.4, to each field. It was easy to differentiate between fields under conventional or organic farming. However, within the sub-sample of fields under conventional farming, crop management strategies “N2a” or “N2c” were poorly represented, and “N0” and “N1” were difficult to differentiate. This is why we used raw data from this survey only to fill in the “Average conventional” and “N3” categories of crop management strategies.

**Fig. 1:**
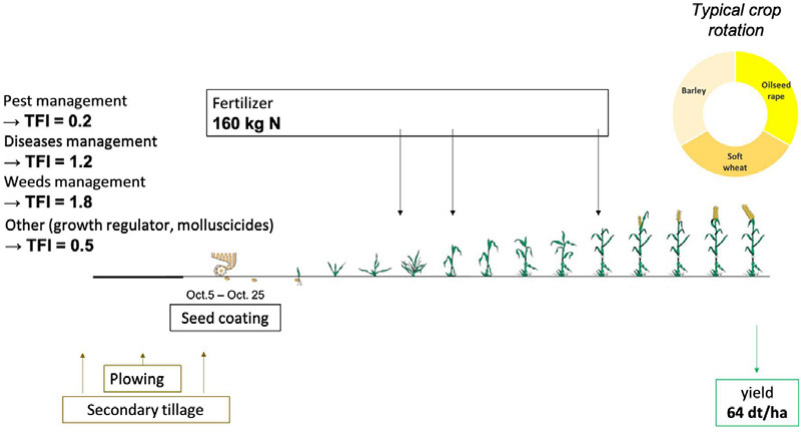
Representation of the typical crop management route for soft wheat, in the intermediate limestone plateau zone, cultivated in a crop sequence with oilseed rape and following an “average conventional” crop management strategy.

We concluded by aggregating the raw data by crop, zone, crop sequence type and crop management strategy (i.e. “Average conventional” if under conventional farming or “N3” if under organic farming). For each cropping situation represented in the aggregated data, we derived a typical crop management route, such as the one illustrated by Fig. 1.

#### 4.2.2 Expert elicitation with agricultural advisors

To complete the “N0”, “N1”, “N2a” and “N2c” and consolidate the “N3” crop management strategies, we individually interviewed 51 experts. These experts were identified by their involvement in the DEPHY FERME network, which has been active for over a decade and now brings together around 2000 farmers. They were mostly agricultural advisors working at local chambers of agriculture or in other agricultural services. We selected them for their day-to-day work with innovative farmers and the expertise they had thus developed regarding what low input crop management strategies look like and how they perform. Interviews were mostly carried out remotely and lasted from one to three hours. They began by an introductive talk explaining the context and framework of our research. We then asked the expert to describe a typical crop management route for each cropping situation in which they are competent. Here, we used the typical crop management routes described in the “*Pratiques culturales grandes cultures et prairies*” field survey raw data as a support for discussion. We questioned the expert as follows: “According to public statistics, a typical crop management route for crop X, in zone Y and in crop sequence Z looks like that.”, at which point we would show them the typical crop management route, “If the crop was managed according to crop management strategy N0, what would have changed? And for N1? N2a?…”. On average, five experts were interviewed for each cropping situations, thus enabling us to take into account the subjectivity of expert knowledge. After the round of individual interviews, we sent the experts the typical crop management routes described for each cropping situations by e-mail for validation and corrected crop management route descriptions according to their reactions.

### 4.3 Indicator computation

#### 4.3.1 Translating crop management routes into quantitative data

Typical crop management route descriptions, as the one illustrated by Fig. 1, were a good support to discuss low-input crop management practices with experts. However, they were inadequate to efficiently compute values for the 30 indicators and 496 cropping situations considered. Thus, we translated these typical crop management routes descriptions into quantitative data, which correspond to the file 1_crop_management_routes.csv, described in Section 2.1. Basically, this step associated each cropping situation with the number and nature of the technical operations it involved and the amount and nature of the input it required and of its yield.

#### 4.3.2 Collecting additional parameters required to compute indicator values

Additional parameters were required to compute economic, environmental and social performances indicators from the quantitative data resulting from the previous step. For example, selling price and input costs were required to calculate profitability from yield and input quantity information. We compiled such parameters values from numerous technical publications from agricultural services, into files 2_crop_parameters.csv, 3_input_parameters.csv and 4_operation_parameters.csv, described in Section 2.2, 3.3, 3.4 respectively.

#### 4.3.3 Computing indicator values from quantitative descriptions of crop management routes

We used quantitative data associated to each cropping situation and additional parameters to compute indicator values, following the equations presented in Section 2.5. In addition to the indicators relating to crop management, we also included 8 indicators relating to livestock farming. These are specific to forage crops (silage corn, temporary grassland and alfalfa) and have been developed using a different approach, due to the complementary nature of crop production and its use by livestock. For different production areas in France, case studies provide a detailed description of the zootechnical functioning of one or more livestock production systems and the associated technical and economic performance. The farming system is associated with a crop rotation that determines the forage calendar. The approach developed allows us to link one or more case-types to our different cropping situations for forage crops and get new indicators. This resulted in 5_indicators_values.csv.

## Limitations

Combining public data analysis and expert knowledge elicitation, we produced data about the economic, environmental and social performances of five crop management strategies showing contrasting degrees of reliance on inputs (i.e. synthetic pesticides and fertilisers).

While our aim was for the spatial validity domain of our dataset to cover the whole agricultural area of metropolitan France, it was in fact limited to arable crops and grasslands, which cover 88 % of that area. [6] Vineyards, orchards and vegetable production were not considered.

The temporal validity domain was limited by the current stage of knowledge regarding low-input crop management practices, as well as by the sociotechnical or biophysical context in which they perform. For example, the economic performances assessed can only represent the range of crops prices or input costs found in recent years, and not how those will evolve in future market conditions.

## Ethics Statement

The authors have read and follow the ethical requirements for publication in Data in Brief and confirm that the work presented here does not involve human subjects, animal experiments, or any data collected from social media platforms.

## Credit Author Statement

**Simon Buresi:** Software, Formal analysis, Investigation, Writing - Original Draft; **Chantal Loyce:** Methodology, Writing - Review & Editing. **Rémy Ballot:** Conceptualisation, Methodology, Writing - Review & Editing, Supervision, Funding acquisition.

## Data Availability

DataverseRéférentiel de performances économiques, environnementales et sociales de pratiques culturales économes en intrants (Original data).
